# Cortically Mediated Muscle Responses to Balance Perturbations Increase with Perturbation Magnitude in Older Adults with and without Parkinson's Disease

**DOI:** 10.1523/ENEURO.0423-25.2026

**Published:** 2026-04-01

**Authors:** Scott E. Boebinger, Aiden M. Payne, Jifei Xiao, Giovanni Martino, Michael R. Borich, J. Lucas McKay, Lena H. Ting

**Affiliations:** ^1^Wallace H. Coulter Department of Biomedical Engineering, Georgia Institute of Technology & Emory University, Atlanta, Georgia 30322; ^2^Department of Physical Therapy, College of Health Sciences and Professions, Ohio University, Athens, Ohio 45701; ^3^Department of Biomedical Sciences, University of Padova, Padua 35131, Italy; ^4^Division of Physical Therapy, Department of Rehabilitation Medicine, Emory University, Atlanta, Georgia 30322; ^5^Department of Neurology, Emory University, Atlanta, Georgia 30322; ^6^Department of Biomedical Informatics, Emory University, Atlanta, Georgia 30322

**Keywords:** aging, balance control, modeling, Parkinson’s disease, sensorimotor transformations

## Abstract

We lack a mechanistic understanding of how cortical contributions to balance control change in aging and Parkinson's disease (PD). Balance is governed by brainstem circuits, with higher-order centers like the cortex or basal ganglia becoming engaged as challenge increases or balance health declines. We previously showed that parallel sensorimotor feedback loops engaging brainstem and cortical circuitry contribute to muscle activity for balance control in young adults (YAs). Here, we analyze data from male and female older adults (OAs) with and without PD, decomposing perturbation-evoked tibialis anterior and medial gastrocnemius muscle activity into hierarchical components based on latencies of feedback control loops. We found that balance-correcting muscle activity followed a stereotypical waveform of long-latency responses (LLRs): LLR1 began ∼120 ms and LLR2 occurred ∼210 ms, respectively, consistent with subcortical and cortical feedback latencies. Both LLRs increased with balance challenge and could be explained by center of mass kinematics. Perturbation-evoked antagonist muscle activity consisted of destabilizing and stabilizing components categorized based on whether they resist the kinematic errors that drive their activation. The destabilizing component occurred at ∼180 ms and was negatively correlated with clinical measures of balance ability in the OA but not PD group. Exploratory comparisons showed OA and PD groups had larger LLR2s at lower challenge levels than YAs, consistent with greater cortical engagement during balance with aging. These findings demonstrate that a neuromechanical model can decompose perturbation-evoked muscle activity into hierarchical components related to clinical balance ability and identify mechanistic changes in the neural control of balance without direct brain measurements.

## Significance Statement

We show that reactive balance recovery in older adults with and without Parkinson's disease can be decomposed into distinct components that reflect hierarchical brainstem, cortical, and basal ganglia feedback loops. Using a neuromechanical model of delayed task-level feedback control, we link these components to perturbation difficulty and clinical balance ability in older adults. This framework connects specific features of agonist and antagonist muscle activity to underlying neural control processes without requiring direct brain recordings. Our findings provide a mechanistic basis for age- and disease-related changes in balance control that can inform individualized assessment and future rehabilitation strategies.

## Introduction

Balance impairments are prevalent in older adults (OAs) and Parkinson's disease (PD) and are associated with less brainstem-mediated control of balance (Park et al., 2015; Li et al., 2018), but contributions from higher-order circuits remain poorly understood. To maintain balance, multisensory information is processed by the nervous system to generate motor commands (Mack et al., 2013). As health declines or challenge increases, the control of balance shifts from brainstem-mediated (Honeycutt et al., 2009) to engage cortical circuits (Jacobs and Horak, 2007; Stuart et al., 2018). Reactive balance recovery is a robust paradigm to investigate shifts in balance control because challenge can be adjusted by varying perturbation size. The discrete nature of the perturbation and temporal resolution of electromyography (EMG) allows for evoked muscle activity to be attributed to different neural circuits based on latency (Reschechtko and Pruszynski, 2020; Boebinger et al., 2024). Here we investigate whether cortical contributions to balance control increase in OAs and PD and determine whether this increase is related to balance ability.

Following externally imposed disturbances, the agonist, or “prime mover,” muscle is activated in a stereotypical sequence of short- and long-latency responses (SLR and LLR, respectively; Pruszynski et al., 2011; Reschechtko and Pruszynski, 2020). SLRs are spinally mediated occurring ∼50 ms postperturbation in the lower limb, respond to muscle stretch, and are not task specific (Carpenter et al., 1999; Ting and Macpherson, 2004). LLRs are mediated by higher-order circuits, occur >100 ms in the lower limb, and contribute to task-level goals regardless of which muscle is stretched (Carpenter et al., 1999; Welch and Ting, 2008, 2009; Reschechtko and Pruszynski, 2020). Individuals with PD exhibit larger LLRs compared with those without (Tatton and Lee, 1975; Rothwell et al., 1983; Scholz et al., 1987). Following a balance perturbation, the magnitude and time course of balance-correcting agonist muscle activity can be reconstructed using a neuromechanical model that scales and sums CoM kinematics by feedback parameters and delays them to account for neural transmission and processing (Welch and Ting, 2008, 2009, 2014). The feedback parameters represent the sensitivity of balance-correcting muscle activation in response to deviations from the desired, upright state (Welch and Ting, 2009, 2014; Boebinger et al., 2024).

A dual-loop neuromechanical model may be able to dissociate balance-correcting responses into hierarchical components without the need for brain recordings, but this has not yet been tested in OAs with or without PD. Recently, we showed that our neuromechanical model can further decompose the LLR into components, LLR1 and LLR2, that arise at latencies consistent with brainstem and cortical circuits, respectively (Boebinger et al., 2024). The LLR1 (∼100 ms postperturbation) was engaged regardless of task difficulty, whereas the LLR2 (∼200 ms postperturbation) became increasingly engaged as task difficulty increased (Boebinger et al., 2024). The LLR2 lags behind perturbation-evoked cortical activity, suggesting the LLR2 may arise from cortical circuits (Boebinger et al., 2024).

CoM feedback can also explain abnormal antagonist muscle activity and identify components that are associated with fall history (McKay et al., 2021). Evoked activity in the antagonist muscle—i.e., the muscle that opposes the action of the agonist muscle—is commonly observed in OAs and individuals with PD, creating cocontraction (Andrews et al., 1972, 1973; Horak et al., 1992; Lang et al., 2019). Aspects of antagonist muscle activity identified using our neuromechanical model are associated with fall history (McKay et al., 2021), therefore we refer to this as the “destabilizing component.” This destabilizing component occurs ∼180 ms after perturbation onset, consistent with basal ganglia circuits (McKay et al., 2021). Following this, the same muscle will also be activated ∼130 ms after to perturbation deceleration which we term the “stabilizing component” since it counteracts perturbation deceleration (McKay et al., 2021). However, we do not know whether the destabilizing component is mediated by similar neural mechanisms as the agonist LLR2 since our prior work used a single feedback loop to reconstruct agonist muscle activity and was unable to capture the agonist LLR2.

**Figure 1.**
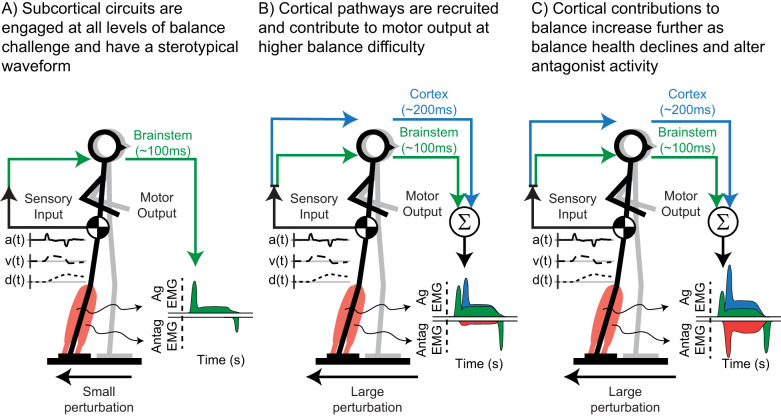
Schematic of hierarchical sensorimotor feedback loops involved in reactive balance control. ***A***, At low levels of balance challenge, we hypothesize that balance control is primarily mediated through brainstem sensorimotor circuits (green) which generate a stereotypical EMG waveform. ***B***, We further hypothesize that at higher levels of balance challenge, higher-order circuits (blue) begin to contribute to balance-correcting muscle activity at longer latencies, leading to alterations in the stereotypical waveform. ***C***, Finally, we hypothesize that as balance health declines, higher-order circuits are further engaged during balance control and lead to abnormal destabilizing antagonist activity (red). Gray stick figure represents the preperturbation stance of the participant, while the black stick figure represents the postperturbation stance of the participant.

Here we dissociated hierarchical components of agonist and antagonist muscle activity in OAs and individuals with PD during reactive balance recovery. We hypothesize that parallel sensorimotor feedback loops engaging brainstem and higher-order circuitry contribute to reactive balance control at different latencies and that the involvement of higher-order circuits increases with challenge, aging, and PD (Fig. 1). We predict that both the LLR1 and LLR2 would increase with perturbation magnitude, but only the LLR2 would be larger in individuals with PD. Finally, we predict that the antagonist destabilizing component and the agonist LLR2 are driven by higher-order neural substrates and occur at similar latencies.

## Materials and Methods

### Ethics statement

All experiments were approved by an Institutional Review Board and all participants gave informed written consent before participating in this experiment.

### Participants

Nineteen OAs and twenty individuals with PD were recruited for this study. Three participants with PD were excluded from this analysis. Two were excluded due to either a brain tumor or severe peripheral neuropathy of the legs noted in their clinical record. The other opted to leave the experiment prior to balance perturbations. After exclusions, 19 older adults (6 female, 71 ± 6 years old, 175 ± 2 cm tall, 79 ± 4 kg; Table 1) and 17 individuals with Parkinson's disease (4 female, 69 ± 6 years old, 171 ± 3 cm tall, 84 ± 6 kg; Table 1) were included in the analyses. Participants were excluded prior to participating if they reported having a history of lower extremity joint pain, contractures, major sensory deficits, evidence of orthopedic, muscular, or physical disability, evidence of vestibular, auditory, or proprioceptive impairment, orthostatic hypotension, and/or any neurological insult other than PD. Participants were recruited from the surrounding community and a local movement disorders clinic through outreach events, word of mouth, flyers, and databases of prior participants from collaborating groups. This work is a secondary analysis of data that has been reported previously (Payne et al., 2021, 2022, 2023).

**Table 1.** Clinical and demographic characteristics of study participants

Participant ID	Age	Gender	Height (cm)	Weight (kg)	miniBEST score (/28)	Hoehn and Yahr stage	MDS UPDRS-III score (/132)	PD duration (years)
OA01	70	M	184.1	85.8	28	–	–	–
OA02	81	F	152.3	54.5	19	–	–	–
OA03	72	F	164.5	62.4	24	–	–	–
OA04	76	M	183.5	83.4	25	–	–	–
OA05	75	M	177	71	24	–	–	–
OA06	73	M	173.5	76.6	24	–	–	–
OA07	75	M	177.5	84.7	27	–	–	–
OA08	82	M	172	74.9	23	–	–	–
OA09	69	M	178	74.8	25	–	–	–
OA10	61	M	184	102.3	23	–	–	–
OA11	67	F	169.3	75.4	27	–	–	–
OA12	66	M	188.6	82.5	22	–	–	–
OA13	68	M	170.8	70.9	25	–	–	–
OA14	65	F	161.6	68.5	26	–	–	–
OA15	67	F	170.7	82.1	24	–	–	–
OA16	63	M	176.5	98.4	27	–	–	–
OA17	62	M	196.4	122.8	24	–	–	–
OA18	81	F	169.4	55.5	25	–	–	–
OA19	76	M	174	70.2	26	–	–	–
PD01	68	F	157.7	65.8	23	II	13	3
PD02	64	M	173.4	116.6	12	IV	48	6
PD03	62	M	171.5	78.8	28	II	14	5
PD04	67	M	180	92.5	27	II	24	3
PD05	75	M	192.3	92.6	25	II	22	3
PD06	69	M	164.5	58	21	III	50	15
PD07	79	F	150.4	57.3	22	II	37	7
PD08	76	M	185.5	95.6	14	IV	47	9
PD09	71	M	170	84.7	26	II	14	8
PD10	66	M	176	80.2	22	II	29	5
PD11	71	M	173.8	107.9	25	II	42	7
PD12	76	F	166	46.9	19	III	21	6
PD13	69	F	154	83	21	II	12	3
PD14	69	M	169.5	77.1	25	II	10	4
PD15	79	M	170.1	57.8	7	V	49	9
PD16	59	M	169.7	146	10	III	47	4
PD17	57	M	183	95.8	27	II	20	7

Note that higher miniBEST scores correspond to better balance performance whereas higher MDS UPDRS-III scores correspond to a greater motor impairment related to Parkinson's disease.

### OFF medications

Individuals with PD were asked to forgo their dopaminergic medications for PD for a minimum of 12 h before participating in this study. Each participant's neurologist was consulted and signed a clearance form prior to participants withholding their medications for this study. Clinical and behavioral measures were collected during this OFF medication session.

### Balance ability

Participant balance ability was determined via the mini Balance Evaluation Systems Test (miniBEST) which assesses sensory orientation, dynamic gait, anticipatory postural control, and reactive postural control (Leddy et al., 2011; Löfgren et al., 2014; Magnani et al., 2020). For items that scored the left and right side separately, only the lower of the two scores was considered which results in a maximum score of 28 (Löfgren et al., 2014), where higher scores indicate better balance ability.

### Parkinson's disease motor symptom severity

For individuals with PD, we examined clinical measures of disease severity to determine if these measures could explain the variance in the hierarchical components identified by our neuromechanical model. The severity of motor impairment in participants with PD was assessed via the motor subscale of the International Parkinson and Movement Disorder Society's Unified Parkinson's Disease Rating Scale (MDS UPDRS-III). This test was administered by A.M.P., who was certified to administer the assessment by the Movement Disorder Society, and video recordings of these assessments were scored by a practicing neurologist.

### Parkinson's disease duration

The number of years since PD diagnosis was self-reported by participants with PD at the time of the study and verified from their clinical records when possible.

### Balance perturbations

**Figure 2.**
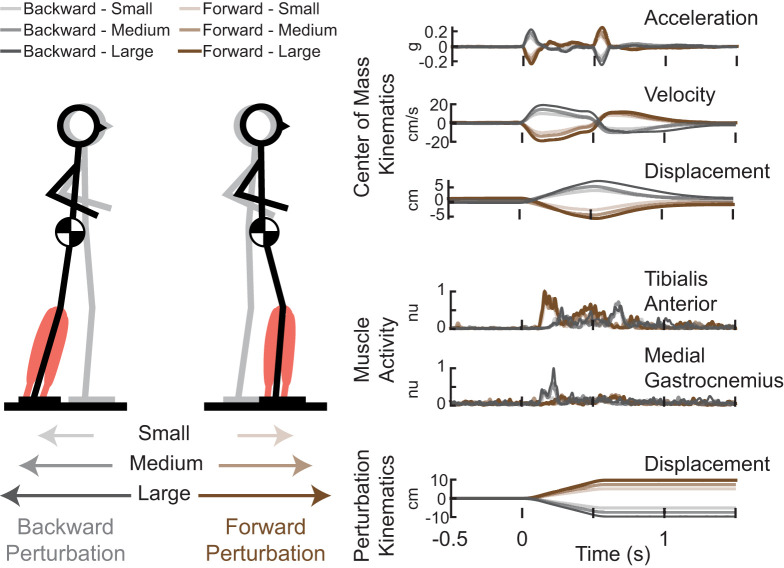
Experimental paradigm. Translational support-surface perturbations were delivered at unpredictable timing, direction, and magnitude. Gray stick figure represents the preperturbation stance of the participant, while the black stick figure represents the postperturbation stance of the participant. Perturbation kinematics, muscle activity, and center of mass kinematics were recorded throughout the perturbation and recovery of balance. Brown shaded time series represent forward support-surface translations, and gray shaded time series represent backward support-surface translations. Darker shading indicates larger perturbation magnitudes.

As previously described (Payne et al., 2021, 2022, 2023), OAs with and without PD stood barefoot with feet shoulder width apart on a motorized platform (Factory Automation Systems) and underwent a series of 48 translational support-surface perturbations that were delivered at unpredictable timing, direction, and magnitude. Each participant received eight perturbations in six different conditions (forward and backward direction at three magnitudes): a small perturbation (5.1 cm, 11.1 cm/s, 0.15 g), which was identical across participants, and two larger magnitudes (medium: 7–7.4 cm, 15.2–16.1 cm/s, and 0.21–0.22 g, and large: 8.9–9.8 cm, 19.1–21.0 cm/s, and 0.26–0.29 g). Medium and large perturbations were adjusted based on participant height to ensure perturbations were mechanically similar across participants (Payne et al., 2021, 2022, 2023; Fig. 2). All perturbation magnitudes had 500 ms between initial acceleration and initial deceleration of the platform. To limit predictability, perturbation magnitude and direction were presented in pseudorandom block order, with each of the eight blocks containing one perturbation of each magnitude and direction. Three different block-randomized orders were used across participants to randomize any effect of trial order (Payne et al., 2021, 2022, 2023). Participants were instructed to keep their arms crossed across their chest, focus their vision on a fixed location ∼4.5 m away, and try to recover balance without taking a step. Trials in which a participant took a step (8.71% of all trials, 9.25% in PD, and 8.21% in OA) were excluded from our analyses because it changes the task-level goal of returning the CoM to the initial posture (Welch and Ting, 2009; Chvatal et al., 2011), precluding the relevance of the CoM feedback hypothesis tested by our delayed sensorimotor response model. A total of five participant × condition pairs were removed from analysis. Four participants (three OA and one PD) had fewer than three nonstepping trials at the largest perturbation magnitude for forward perturbations whereas one PD participant had fewer than three stepping trials at the largest perturbation in the backward direction. Successful nonstepping trials were identified using platform-mounted force plates (AMTI OR6-6). If there were under three successful nonstepping trials averaged for a certain participant-condition pair, it was removed from analyses, as a minimum of three trials is necessary for robust CoM feedback model fitting (Welch and Ting, 2014). To limit fatigue, 5 min breaks were given every 15 min of experimentation or more frequently if requested. The duration of the perturbation series was 21 ± 2 min (PD: 20 ± 1 min; OA: 21 ± 2 min; Payne et al., 2022).

### Center of mass kinematics

Kinematic marker data were collected at 100 Hz and synchronized using a 10-camera Vicon Nexus 3D motion analysis system (Vicon). Body segment kinematics were determined from a custom marker set covering head–arms–trunk, thigh, shank, and foot segments. Center of mass (CoM) displacement was derived from kinematic data as a weighted sum of segmental masses. CoM velocity was taken as the derivative of CoM displacement after smoothing using a third-order Savitzky–Golay filter with a filter size of 48 samples (Safavynia and Ting, 2013; Fig. 2). CoM acceleration was computed from ground reaction forces obtained by the platform-mounted force plates (AMTI OR6-6), divided by participant mass (Fig. 2). Peak CoM excursion was calculated as the maximum displacement of the CoM relative to the base of support following the balance perturbation.

### Electromyography

Surface EMGs (Motion Analysis Systems) were collected bilaterally from the tibialis anterior (TA) and medial gastrocnemius muscle (MG) muscles (Fig. 2). Analysis focused on the left TA and MG since this agonist–antagonist muscle pair are activated in forward and backward support-surface perturbations. Prior to EMG electrode placement, skin was shaved if necessary and scrubbed with an isopropyl alcohol wipe. EMG electrodes were placed using standard procedures (Basmajian, 1980). Bipolar silver silver-chloride electrodes were used (Norotrode 20, Myotronics). Electromyography signals were sampled at 1,000 Hz and anti-alias filtered with an online 500 Hz low-pass filter. Raw EMG signals were then high-pass filtered at 35 Hz offline with a sixth-order zero-lag Butterworth filter, mean-subtracted, half-wave rectified, and subsequently low-pass filtered at 40 Hz (Welch and Ting, 2014; Payne and Ting, 2020; McKay et al., 2021). EMG signals were epoched between −200 and 1,200 ms relative to perturbation onset. Single-trial EMG data were normalized to a maximum value of 1 across all trials within each participant. EMG data were then averaged across trials within each perturbation magnitude for each participant.

### Neuromechanical models

Perturbation-evoked agonist and antagonist muscle activity were reconstructed using a series of delayed feedback models to investigate the relationship between sensory information and muscle activity. All neuromechanical models employed here use kinematic signals of balance error, defined as CoM displacement (d), velocity (v), and acceleration (a) relative to the base of support, as predictors to reconstruct perturbation-evoked muscle activity (McKay et al., 2021; Boebinger et al., 2024).

### Agonist neuromechanical model

**Figure 3.**
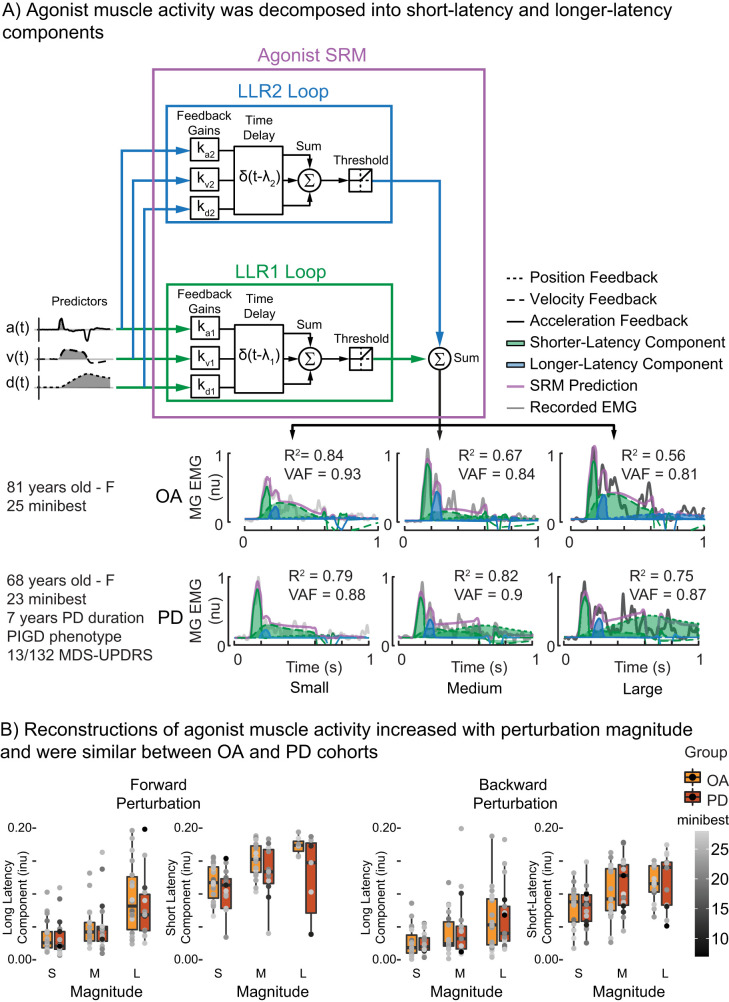
Agonist muscle activity was decomposed into different hierarchical components based on latency. ***A***, Activity of the agonist muscle, defined as the muscle initially lengthened by the perturbation (medial gastrocnemius for the backward perturbation shown here) was decomposed into LLR1 (green) or LLR2 (blue) components. Agonist muscle activity was reconstructed as the weighted sum of positive CoM kinematics (those that stretch the muscle) that was delayed by two separate time delays reflecting brainstem (λ_1_) or cortical (λ_2_) circuits. ***B***, Group summary of the integrated output of the LLR1 and LLR2 feedback loops for forward and backward perturbations.

The agonist neuromechanical model uses two feedback loops with different loop delays to account for the inherently different latencies between brainstem-mediated and cortically mediated muscle activity as previously validated in YAs (Boebinger et al., 2024; Fig. 3). This model is used to decompose perturbation-evoked activity in the agonist muscle, defined as the muscle initially stretched by the perturbation, into components attributed to different neural circuits based on latency.

The LLR1 feedback loop multiplies the CoM kinematic predictors that stretch the agonist muscle by their respective feedback gains (*k*_d1_, *k*_v1_, *k*_a1_). These weighted signals are then summed, and delayed by a time delay (λ_1_) to account for ascending and descending neural transmission and processing (Eq. 1):

$$\mathrm{em}g_{1}(t)∼\lfloor k_{d1}\cdot d_{\mathrm{CoM}}(t-\lambda_{1})+\;k_{v1\;}\cdot v_{\mathrm{CoM}}(t-\lambda_{1})+\;k_{a1}\cdot a_{\mathrm{CoM}}(t-\lambda_{1})\rfloor .$$

The output from the LLR1 feedback loop [emg_1_(*t*)] was half wave rectified with a threshold value of 0 to represent the overall non-negative net output to motor pools (Eq. 2; McKay et al., 2021; Boebinger et al., 2024):

⌊⋅⌋=max(0,⋅).

The longer-latency feedback loop uses the same predictors that stretch the agonist muscle as the brainstem feedback loop but weights these kinematic signals by independent feedback gains (*k_d_*_2_, *k_v_*_2_, *k_a_*_2_) and is delayed by a longer time delay (λ_2_) to account for the longer latency required for ascending and descending neural transmission and processing in cortical circuits (Eq. 3):

$$\mathrm{em}g_{2}(t)∼\lfloor k_{d2}\cdot d_{\mathrm{CoM}}(t-\lambda_{2})+\;k_{v2}\cdot v_{\mathrm{CoM}}(t-\lambda_{2})+\;k_{a2}\cdot a_{\mathrm{CoM}}(t-\lambda_{2})\rfloor .$$

The output from the longer-latency feedback loop [emg_2_(*t*)] was also half wave rectified with a threshold value of 0 to represent the overall net output to motor pools (Eq. 2; McKay et al., 2021; Boebinger et al., 2024).

The output from both feedback loops were then summed linearly in line with previous modeling approaches of similar behavior in the upper limb (Pruszynski et al., 2011; Eq. 4):

$$\mathrm{em}g_{\mathrm{agonist}}(t)=\mathrm{em}g_{1}(t)+\mathrm{em}g_{2}(t).$$

### Antagonist neuromechanical model

**Figure 4.**
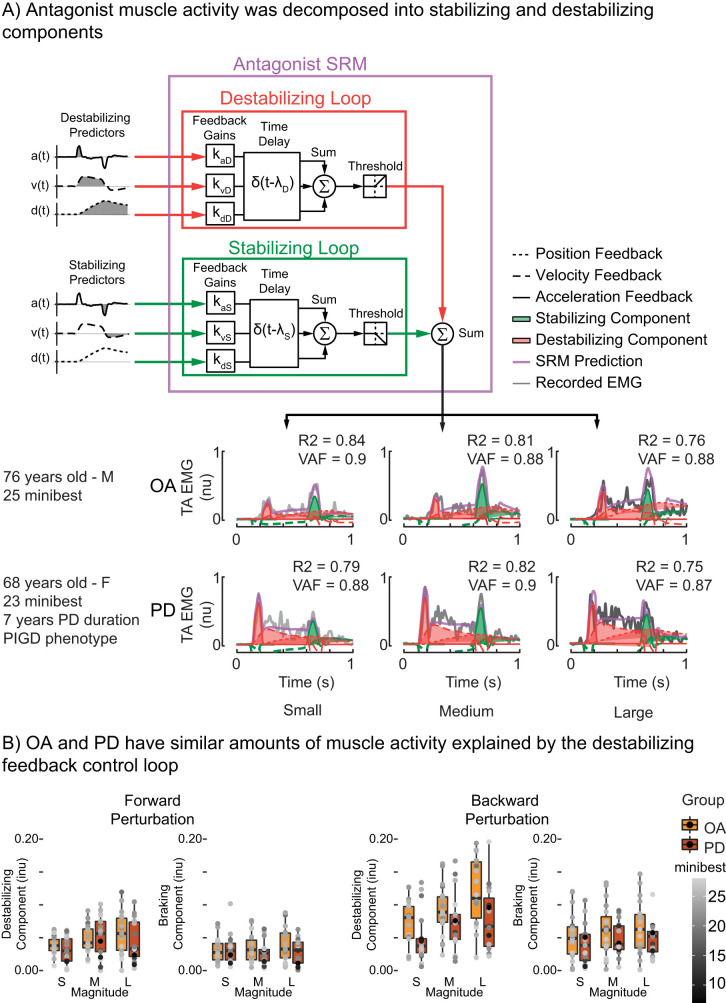
Antagonist muscle activity was decomposed into stabilizing and destabilizing components. ***A***, Activity of the antagonist muscle, defined as the muscle initially shortened by the perturbation, was decomposed into hierarchical components based on latency using the antagonist neuromechanical model. ***B***, Group summary of the integrated output of the stabilizing and destabilizing feedback loops for forward and backward perturbations.

The antagonist neuromechanical model also uses two feedback loops with different delays to reconstruct antagonist muscle activity and was developed in a prior study on a separate PD group (McKay et al., 2021; Fig. 4). This model was used to decompose perturbation-evoked antagonist muscle activity into stabilizing and destabilizing components.

The stabilizing feedback loop multiplies CoM predictors that stretch the antagonist muscle by their respective feedback gains (*k*_dS_, *k*_vS_, *k*_aS_). These weighted signals are then summed and delayed by a common time delay (λ_s_) to account for ascending and descending neural transmission and processing (Eq. 5):

$$\mathrm{em}g_{S}(t)∼\lfloor k_{\mathrm{dS}}\cdot d_{\mathrm{CoM}}(t-\lambda_{s})+\;k_{\mathrm{vS}\;}\cdot v_{\mathrm{CoM}}(t-\lambda_{s})+\;k_{\mathrm{aS}}\cdot a_{\mathrm{CoM}}(t-\lambda_{s})\rfloor .$$

The output from the stabilizing feedback loop [emg_stabilzing_(*t*)] is half wave rectified with a threshold value of 0 (Eq. 2).

The destabilizing feedback loop uses CoM predictors that shorten the antagonist muscle [−*a*(*t*), −*v*(*t*), and −*d*(*t*)] and multiplies these kinematic signals by separate feedback gains (*k*_dD_, *k*_vD_, *k*_aD_). These weighted signals are then summed and delayed by a common time delay (λ_D_) to account for ascending and descending neural transmission and processing (Eq. 6):

$$\mathrm{em}g_{D}(t)∼\lfloor -k_{\mathrm{dD}}\cdot d_{\mathrm{CoM}}(t-\lambda_{D})-\;k_{\mathrm{vD}}\cdot v_{\mathrm{CoM}}(t-\lambda_{D})-\;k_{\mathrm{aD}}\cdot a_{\mathrm{CoM}}(t-\lambda_{D})\rfloor .$$

The output from the destabilizing [emg_D_(*t*)] feedback loop was also half wave rectified with a threshold value of 0 to represent the overall net output to motor pools (Eq. 2; McKay et al., 2021). We further decomposed the output from this destabilizing feedback loop into component parts attributed to the CoM acceleration feedback component (*k*_aD_) and the CoM velocity and displacement feedback component (*k*_vD_ + *k*_dD_).

The output from the stabilizing and destabilizing feedback loops were then summed to reconstruct the antagonist muscle (Eq. 7).

$$\mathrm{em}g_{\mathrm{antagonist}}(t)∼\mathrm{em}g_{S}(t)+\mathrm{em}g_{D}(t).$$

### Model parameter identification

Parameters for both the agonist and antagonist neuromechanical models were selected by minimizing the error between recorded EMG data and the model reconstruction. The reconstruction error was quantified as the sum of the sum of the squared errors of the whole time series as well as the maximum error observed at any sample (Eq. 8):

$$\underset{k_{i},\;\lambda_{i}}{\min}\;\left\{\mu_{s}\int_{t_{\mathrm{start}}}^{t_{\mathrm{end}}}e^{2}dt+\mu_{m}\max(\left|e\right|)+\mu_{k}k^{T}k\right\}.$$

The first term penalizes squared error (*e*^2^) between the recorded data and the model prediction weighted by *μ*_s_. The second term penalizes the maximum error observed between the recorded data and the model reconstruction weighted by *μ*_m_. The final term penalizes the magnitude of the gain parameters (*k*_i_) with weight *μ*_k_ in order to minimize feedback parameters that do not contribute to reconstruction accuracy. The ratio of weights *μ*_s_:*μ*_m_:*μ*_k_ was 1:1:1 × 10^−6^. All optimizations were performed in Matlab 2022b (MathWorks) using the interior-point algorithm in *fmincon.m*, as described previously in literature (McKay et al., 2021; Boebinger et al., 2024).

Separate optimizations were performed to identify model-specific feedback parameters for the individual feedback loops (Eqs. 1, 3, 5, 6). The output from these optimizations are then used as an initial guess when optimizing for double-looped models (Eqs. 4, 7) similar to what was done in prior implementations of this model (McKay et al., 2021; Boebinger et al., 2024). This allows for modifications to the single-loop optimization's output, as model parameters may have been overestimated in order to fit muscle activity that is better explained by the other feedback loop. Lower and upper bounds for the gain parameters were ±10% of the initial guess values, and lower and upper bounds for the delay parameters were ±10 ms of the initial guess values. In all cases, additional parameters supplied to *fmincon.m* were as follows: *TolX*, 1 × 10^−9^; *MaxFunEvals*, 1 × 10^5^; *TolFun*, 1 × 10^−7^, with remaining parameters set to default (McKay et al., 2021; Boebinger et al., 2024).

### Goodness of fit

The goodness of fit for all neuromechanical model reconstructions were assessed using a coefficient of determination (*R*^2^) and variability accounted for (VAF). *R*^2^ was calculated using *regress.m*, a built-in function in Matlab 2022b (MathWorks). VAF was defined as 100*[the square of Pearson's uncentered correlation coefficient], as performed in previous studies (Safavynia and Ting, 2013; McKay et al., 2021; Boebinger et al., 2024).

### Statistical analysis

The integrated area under the curve of each neuromechanical model component (i.e., each feedback control loop; Eqs. 1, 3, 5, 6) was calculated via numerical integration using *trapz.m*, a built-in function in Matlab 2022b (MathWorks). Statistical tests were performed in RStudio version 1.4.1717 (R Core Team). Comparisons of integrated components and CoM excursion between perturbation magnitude and group were performed using a linear mixed-effects model with an interaction between the fixed factors of perturbation magnitude and group with participant as a random factor. Satterthwaite's correction was applied, and post hoc comparisons were performed via comparisons of the estimated marginal means between perturbation magnitudes or group. Correlations between integrated components and clinical measures (miniBEST and MDS UPDRS-III scores) were performed using linear mixed-effects models with interactions between the fixed factors of clinical score and perturbation magnitude with participant as a random factor. Separate linear mixed models were applied for the OA and PD groups. An independent samples *t* test was performed to evaluate whether there were differences in miniBEST scores between PD and OA groups. Comparisons of stepping behavior (step vs no-step) between perturbation magnitude, perturbation direction, and group were performed using a generalized linear mixed-effects model including fixed effects of group, perturbation magnitude, and perturbation direction and all interactions, with participant included as a random intercept. To evaluate whether stepping behavior changed across the session, trial number was included as an additional fixed effect in a separate generalized linear mixed-effects model with participant included as a random intercept. Post hoc comparisons were performed via comparisons of the estimated marginal means. All tests were considered statistically significant at *p* ≤ 0.05. Due to the exploratory nature of the analysis, no a priori sample size calculations or multiple-comparisons corrections were performed.

### Code accessibility

The custom code used for neuromechanical modeling, figure generation, and statistical analysis is freely available on GitHub via https://github.com/Neuromechanics-Lab/HOA_PD_SRM.

## Results

### Balance performance

Individuals with PD had lower clinical scores of balance ability (miniBEST) compared with OAs (*t*_(19.28)_ = 2.4, *p* = 0.027). However, there was no significant difference in peak CoM excursion following a balance perturbation between OAs and individuals with PD for either forward (*F*_(1,33.8)_ = 2.88, *p* = 0.10) or backward perturbations (*F*_(1,34)_ = 0.36, *p* = 0.55). There was also no significant difference in the percentage of trials where a participant took a step between the PD group (9.25% of trials) and the OA group (8.21% of trials; *p* = 0.70). The number of stepping responses also decreased across trials (*p* < 0.001) with no difference between groups (*p* = 0.62).

### Agonist sensorimotor response to perturbations

Parallel sensorimotor feedback loops that independently respond to CoM kinematic errors at different latencies can reconstruct balance-correcting muscle activity in OAs with and without PD. When using the double-loop instead of a single-loop model, model reconstruction accuracy increased by ∼10% (Table 2). The double-loop model had higher reconstruction accuracies compared with the single-loop model in both perturbation directions (Forward: *R*^2^: *t*_(34.5)_ = 11.2, *p* < 0.0001; VAF: *t*_(34.7)_ = 11.0, *p* < 0.0001; Backward: *R*^2^: *t*_(35)_ = 6.6, *p* < 0.0001; VAF: *t*_(35)_ = 6.3, *p* < 0.0001).

**Table 2.** Reconstruction accuracies for single-loop and double-loop agonist neuromechanical model

Model type	Older adults	Individuals with Parkinson's disease
Single loop Model	*R*^2^ = 0.64 ± 0.01	*R*^2^ = 0.61 ± 0.02
	VAF = 0.74 ± 0.01	VAF = 0.72 ± 0.01
Double loop Model	*R*^2^ = 0.74 ± 0.01	*R*^2^ = 0.71 ± 0.01
	VAF = 0.83 ± 0.01	VAF = 0.81 ± 0.01

Longer-latency agonist muscle activity associated with the LLR1 and LLR2 can be explained by CoM kinematic feedback at delays consistent with brainstem and cortical feedback loops, respectively, in OAs with and without PD (Fig. 3*A*). Agonist muscle activity associated with LLR1 was reconstructed using feedback delays of 119 ms ± 1.6 ms for OAs and 116 ms ± 1.3 ms for individuals with PD (Fig. 3*B*). There was no difference in LLR1 latency between groups in either direction (Forward: *F*_(1,34)_ = 1.87, *p* = 0.18; Backward: *F*_(1,34)_ = 0.02, *p* = 0.89). Agonist muscle activity associated with the LLR2 was reconstructed by sensorimotor feedback at delays of 200 ms ± 3.2 ms in OAs and 215 ms ± 3.9 ms in individuals with PD. There was a difference in LLR2 latency between groups in only backward perturbations (Forward: *F*_(1,33.9)_ = 0.46, *p* = 0.50; Backward: *F*_(1,34)_ = 4.17, *p* = 0.049).

We further show that LLR1 and LLR2 components of agonist muscle activity increase with balance task difficulty in OAs with and without PD. The LLR1 component of the neuromechanical model increased with perturbation magnitude (Fig. 3*B*) in both perturbation directions (Forward: *F*_(2,65.8)_ = 36.24, *p* < 0.001; Backward: *F*_(2,68)_ = 25.98, *p* < 0.001) but did not differ between groups (Forward: *F*_(1,34.6)_ = 0.37, *p* = 0.55; Backward: *F*_(1,34)_ = 0.31, *p* = 0.58). There were no interactions between perturbation magnitude and group (Forward: *F*_(2,65.8)_ = 1.36, *p* = 0.26; Backward: *F*_(2,68)_ = 0.22, *p* = 0.81). The LLR2 magnitude increased in both groups (Forward: *F*_(2,64.7)_ = 37.6, *p* < 0.001; Backward: *F*_(2,68)_ = 16.97, *p* < 0.001), with no difference between groups (Forward: *F*_(1,34.6)_ = 0.08, *p* = 0.78; Backward: *F*_(1,34)_ = 0.12, *p* = 0.73). There were no interactions between perturbation magnitude and group (Forward: *F*_(2,64.7)_ = 0.06, *p* = 0.94; Backward: *F*_(2,68)_ = 0.34, *p* = 0.71).

### Antagonist sensorimotor response to perturbations

Perturbation-evoked antagonist muscle activity could also be explained by sensorimotor transformations of CoM kinematics via a double-looped neuromechanical model (Fig. 4*A*). The destabilizing component of antagonist muscle (Fig. 4*A*, red trace) at the beginning of the perturbation was reconstructed based on a positive relationship to CoM kinematic errors, i.e., further contributing to these errors, and the subsequent stabilizing component (Fig. 4*A*, green trace) at the end of the perturbation was driven by the perturbation deceleration using a negative relationship to CoM kinematic errors.

The destabilizing component of the antagonist muscle (Fig. 4*A*, red trace) increased in amplitude with perturbation magnitude in both OA and PD groups (Forward: *F*_(2,63.8)_ = 10.15, *p* < 0.001; Backward: *F*_(2,68)_ = 44.12, *p* < 0.001; Fig. 4*B*). There were no differences in the effect of perturbation magnitude between the OA and PD groups in either perturbation direction (Forward: *F*_(1,34.1)_ = 0.30, *p* = 0.59; Backward: *F*_(1,34)_ = 2.29, *p* = 0.14; Fig. 4*B*). There were no interactions between perturbation magnitude and group (Forward: *F*_(2,63.8)_ = 1.59, *p* = 0.21; Backward: *F*_(2,68)_ = 0.39, *p* = 0.68).

Individuals with PD exhibited a larger acceleration-driven component of destabilizing antagonist muscle activity compared with OAs. Individuals with PD had larger integrated acceleration feedback component (Fig. 4*A*, shaded area of the solid red line) compared with OAs in forward perturbations (Forward: *t*_(34)_ = −2.05, *p* = 0.048; Backward: *t*_(34)_ = 1.174, *p* = 0.25). This was also reflected in the destabilizing acceleration feedback gain (*k*_aD_) value itself (Forward: *t*_(34)_ = −2.5, *p* = 0.017; Backward: *t*_(34)_ = 0.72; *p* = 0.48). There was no difference between OA and PD groups in the contribution of the integrated velocity and displacement feedback component (Fig. 4*A*, shaded area of the dotted red lines) in either perturbation direction (Forward: *t*_(34)_ = 0.86, *p* = 0.40; Backward: *t*_(34)_ = 1.37, *p* = 0.18).

Contributions from the stabilizing feedback loop to antagonist muscles (Fig. 4*A*, green trace) that occurred in response to the deceleration of the ramp-and-hold perturbations increased with perturbation magnitude, but only the backward perturbation direction (Forward: *F*_(1,63.9)_ = 0.15, *p* = 0.86; Backward: *F*_(2,68)_ = 3.98, *p* = 0.023; Fig. 4*B*). There was no effect of group on the output from the stabilizing feedback loop (Forward: *F*_(1,33.9)_ = 0.95, *p* = 0.34; Backward: *F*_(1,34)_ = 2.20, *p* = 0.15; Fig. 4*B*). There were no interactions between perturbation magnitude and group (Forward: *F*_(2,63.9)_ = 1.41, *p* = 0.25; Backward: *F*_(2,68)_ = 0.21, *p* = 0.81).

### Destabilizing antagonist activity occurred at latencies consistent with basal ganglia mediation

The onset of the antagonist destabilizing feedback loop occurred at an intermediate latency between that of the agonist muscle LLR1 and LLR2. The antagonist destabilizing cocontraction had an onset latency (λ_D_) of 181 ± 3.6 ms in OAs and 173 ± 2.9 ms for individuals with PD, no difference between groups was found (Forward: *t*_(34)_ = 1.76, *p* = 0.09; Backward: *t*_(34)_ = −0.80, *p* = 0.43). The onset of the antagonist stabilizing muscle activity in response to perturbation deceleration occurred at 135 ± 3.3 ms in OAs and 130 ± 2.2 ms in people with PD.

### Correlations to clinical measures

**Figure 5.**
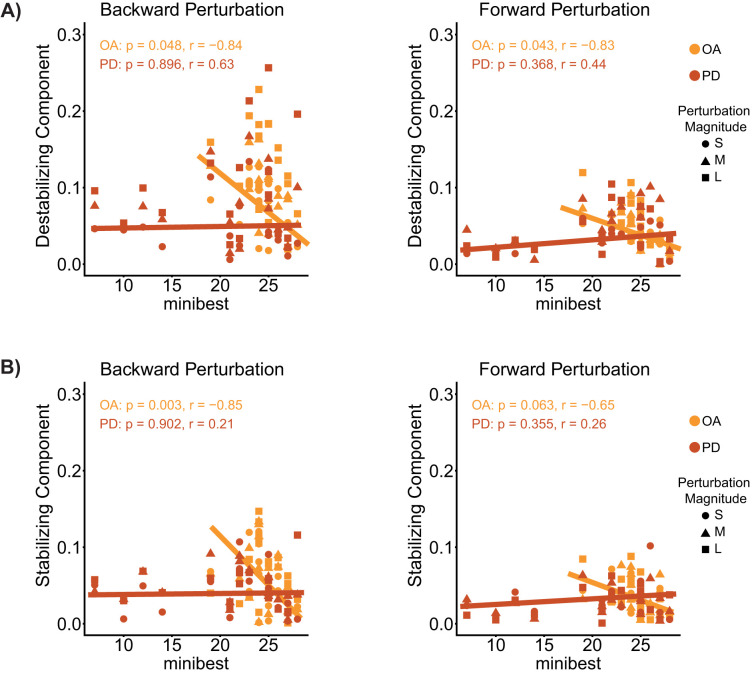
Correlations of components of antagonist muscle activity identified by our neuromechanical model and clinical measures of balance ability (miniBEST). ***A***, The destabilizing component (Fig. 4*A*, red trace). ***B***, The stabilizing component (Fig. 4*A*, green trace).

In the OA group, a greater output from the antagonist destabilizing feedback loop (Fig. 4*A*, red traces) was correlated with lower clinical measures of balance ability (miniBEST) in both perturbation directions (Table 3). In contrast, in the PD group there were no correlations between the integrated component from the antagonist destabilizing feedback loop (Fig. 4*A*, red traces) and higher clinical measures of balance ability (miniBEST; Table 3, Fig. 5).

**Table 3.** Statistical comparisons of neuromechanical model outputs and miniBEST scores

Integrated component	Correlation to miniBEST	Correlation to miniBEST
	Older adults	Parkinson’s disease
Agonist LLR1	Forward: *R*^2^ = 0.71, *p* = 0.50	Forward: *R*^2^ = 0.29, *p* = 0.73
	Backward: *R*^2^ = 0.5, *p* = 0.08̇	Backward: *R*^2^ = 0.34, *p* = 0.55
Agonist LLR2	Forward: *R*^2^ = 0.52, *p* = 0.63	Forward: *R*^2^ = 0.53, *p* = 0.55
	Backward: *R*^2^ = 0.33, *p* = 0.25	Backward: *R*^2^ = 0.24, *p* = 0.91
Antagonist stabilizing	Forward: *R*^2^ = 0.42, *p* = 0.06̇	Forward: *R*^2^ = 0.15, *p* = 0.23
	Backward: *R*^2^ = 0.73, *p* < 0.01*	Backward: *R*^2^ = 0.04, *p* = 0.9
Antagonist destabilizing	Forward: *R*^2^ = 0.67, *p* = 0.043*	Forward: *R*^2^ = 0.19, *p* = 0.37
	Backward: *R*^2^ = 0.70, *p* = 0.048*	Backward: *R*^2^ = 0.40, *p* = 0.90
Antagonist destabilizing (*k*_aD_ component)	Forward: *R*^2^ = 0.06, *p* = 0.98	Forward: *R*^2^ = 0.38, *p* = 0.10
	Backward: *R*^2^ = 0.08, *p* = 0.63	Backward: *R*^2^ = 0.15, *p* = 0.74
Antagonist destabilizing (*k*_vD_ + *i*_dD_ component)	Forward: *R*^2^ = 0.65, *p* = 0.09̇	Forward: *R*^2^ = 0.37, *p* = 0.22
	Backward: *R*^2^ = 0.66, *p* = 0.06̇	Backward: *R*^2^ = 0.40, *p* = 0.92

* indicates *p* values <0.05. ˙ indicates *p* values <0.1.

Interestingly, there was also a negative correlation between output of the stabilizing feedback loop (Fig. 4*A*, green traces) in the antagonist muscle and miniBEST scores in OA, but not PD (Table 3, Fig. 5). That is, OAs who exhibited less muscle activity in response to perturbation deceleration tended to have better clinical measures of balance ability.

Assessments of PD motor symptom severity (MDS UPDRS-III score, PD phenotype, PIGD-subscore, Hoehn and Yahr state) and PD duration could not explain the variance in any of the components identified by our neuromechanical models, except for Hoehn and Yahr scores and the agonist LLR1 component in the forward direction (Table 4).

**Table 4.** Statistical comparisons of neuromechanical model outputs and Parkinson's disease specific clinical measures

Integrated component	MDS UPDRS-III	PD phenotype	PIGD-subscore	Hoehn and Yahr state	PD duration
Agonist LLR1	Forward: *p* = 0.85 Backward: *p* = 0.84	Forward: *F*_(2,14.1)_ = 0.4, *p* = 0.68 Backward: *F*_(2,14)_ = 1.16, *p* = 0.34	Forward: *F*_(6,9.53)_ = 2.02, *p* = 0.16 Backward: *F*_(6,10)_ = 0.85, *p* = 0.56	Forward: *F*_(3,12.3)_ = 3.5, ***p*** **=** 0.049* Backward: *F*_(3,13)_ = 0.17, *p* = 0.92	Forward: *p* = 0.87 Backward: *p* = 0.89
Agonist LLR2	Forward: *p* = 0.46 Backward: *p* = 0.97	Forward: *F*_(2,14.2)_ = 0.4, *p* = 0.68 Backward: *F*_(2,14)_ = 1.0, *p* = 0.38	Forward: *F*_(6,10.1)_ = 1.26, *p* = 0.36 Backward: *F*_(6,10)_ = 0.51, *p* = 0.79	Forward: *F*_(3,12.8)_ = 1.0, *p* = 0.41 Backward: *F*_(3,13)_ = 0.5, *p* = 0.69	Forward: *p* = 0.83 Backward: *p* = 0.83
Antagonist stabilizing	Forward: *p* = 0.19 Backward: *p* = 0.75	Forward: *F*_(2,14.2)_ = 1.21, *p* = 0.33 Backward: *F*_(2,14)_ = 0.29, *p* = 0.75	Forward: *F*_(6,9.41)_ = 0.84, *p* = 0.57 Backward: *F*_(6,10)_ = 0.55, *p* = 0.76	Forward: *F*_(3,12.8)_ = 0.37, *p* = 0.77 Backward: *F*_(3,13)_ = 0.17, *p* = 0.91	Forward: *p* = 0.59 Backward: *p* = 0.29
Antagonist destabilizing	Forward: *p* = 0.59 Backward: *p* = 0.44	Forward: *F*_(2,13.9)_ = 0.3, *p* = 0.74 Backward: *F*_(2,14)_ = 0.15, *p* = 0.86	Forward: *F*_(6,9.92)_ = 1.31, *p* = 0.34 Backward: *F*_(6,10)_ = 0.1, *p* = 0.995	Forward: *F*_(3,12.8)_ = 1.2, *p* = 0.35 Backward: *F*_(3.13)_ = 0.10, *p* = 0.96	Forward: *p* = 0.75 Backward: *p* = 0.23
Antagonist destabilizing (k_aD_ component)	Forward: *p* = 0.90 Backward: *p* = 0.39	Forward: *F*_(2,13.8)_ = 0.55, *p* = 0.59 Backward: *F*_(2,14)_ = 1.05, *p* = 0.38	Forward: *F*_(6,9.42)_ = 0.74, *p* = 0.63 Backward: *F*_(6,10)_ = 0.66, *p* = 0.69	Forward: *F*_(3,12.6)_ = 0.69, *p* = 0.57 Backward: *F*_(3,13)_ = 0.28, *p* = 0.84	Forward: *p* = 0.47 Backward: *p* = 0.37
Antagonist destabilizing (*k*_vD_ + *k*_dD_ component)	Forward: *p* = 0.59 Backward: *p* = 0.50	Forward: *F*_(2,14)_ = 0.34, *p* = 0.72 Backward: *F*_(2,14)_ = 0.27, *p* = 0.77	Forward: *F*_(6,9.97)_ = 1.31, *p* = 0.34 Backward: *F*_(6,10)_ = 0.07, *p* = 0.998	Forward: *F*_(3,12.9)_ = 1.14, *p* = 0.37 Backward: *F*_(3,13)_ = 0.1, *p* = 0.96	Forward: *p* = 0.75 Backward: *p* = 0.24

## Discussion

Our data show that parallel sensorimotor feedback loops engaging hierarchical neural circuits can explain perturbation-evoked agonist and antagonist muscle activity during reactive balance recovery in older adults with and without Parkinson's disease. We provide experimental and computational evidence that agonist muscle activity can be broken into separate components that occur at latencies consistent with brainstem-mediated (LLR1) and cortically mediated (LLR2) feedback loops. This result further supports our hypothesis that parallel sensorimotor feedback loops engaging brainstem and higher-order circuits contribute to reactive balance control at different latencies (Boebinger et al., 2024). Therefore, our approach may provide a mechanistic assessment of cortical engagement during transient reactive balance responses without requiring measurement of brain activity. Further, abnormal antagonist muscle activity can be explained by sensorimotor transformations at delays consistent with basal ganglia circuits, which may contribute to impaired balance (McKay et al., 2021). Overall, we show that hierarchical relationships between balance perturbations and evoked muscle activity likely reflect the activity of multiple distinct neural circuits that underlie balance function.

Our neuromechanical model enables us to dissociate the contributions of hierarchical neural circuits to evoked muscle activity, at latencies consistent with prior studies. The initial burst of agonist muscle activity (i.e., the start of LLR1) occurred at latencies ∼120 ms, consistent with those previously observed (Welch and Ting, 2008; 2009, 2014; McKay et al., 2021; Boebinger et al., 2024). The LLR1 is considered to be mediated by brainstem sensorimotor circuits (Jacobs and Horak, 2007) as they persist in decerebrate animals (Honeycutt et al., 2009) and contribute to task-level goals, such as endpoint regulation and CoM stabilization, regardless if the muscle is stretched (Taube et al., 2006; Welch and Ting, 2008, 2009; Pruszynski et al., 2011; Pruszynski and Scott, 2012; Reschechtko and Pruszynski, 2020). The subcortical basis of muscle activity in LLR1 is further supported by the fact that perturbation-evoked cortical activity occurs at similar latencies as the LLR1 and therefore cannot contribute to it (Payne et al., 2019; Payne and Ting, 2020; Boebinger et al., 2024). However, second burst of agonist muscle activity (i.e., LLR2) could can be cortically mediated as it occurs at latencies ∼200 ms (Boebinger et al., 2024), and the LLR2 can be altered by transcranial magnetic stimulation, while the LLR1 cannot (Petersen et al., 1998; Taube et al., 2006).

Contrary to our hypothesis that people with PD engage greater cortical resources for balance control (Jacobs and Horak, 2007; Reschechtko and Pruszynski, 2020), we found similar longer-latency muscle activity in OAs with and without PD. Prior work shows that individuals with PD tend to exhibit increased in cortical activity (Park et al., 2015; Stuart et al., 2018, 2019) and exhibit larger LLRs following upper limb perturbation compared with individuals without PD (Lee and Tatton, 1975; Tatton and Lee, 1975; Rothwell et al., 1980, 1983; Scholz et al., 1987). However, we found that OAs with PD had similar LLR2 components, which may be due to experimental differences in testing functional LLRs in the lower limb during automatic postural responses versus during isometric upper limb tasks. The lack of group differences in LLR2 cannot be attributed to normalization of the EMG across individuals as similar results were obtained when LLR2 was measured as a fraction of total EMG. The lack of difference across groups may arise because our approach dissociates LLR2 into a cortical component due to acceleration feedback that is summed with slower subcortical components, rather than taking the entire amplitude of the LLR2 initial burst, thereby dissociating contributions arising at latencies consistent with cortical control of balance (Jacobs and Horak, 2007; Pruszynski et al., 2011; Reschechtko and Pruszynski, 2020). Additionally, LLR2 only captures the earliest feedback component where cortical circuits could contribute to balance control and does not consider later descending contributions that are necessary for a change of balance strategy such as taking a step (Brauer et al., 2002; Chvatal et al., 2011; Solis-Escalante et al., 2020).

Considering the activity of antagonist muscles in balance may be critical to assessing the relationship between balance control and balance ability. Consistent with our prior findings, the antagonist activity had latencies consistent with basal ganglia involvement in both OA and PD groups, with latencies of ∼180 ms, which is after reticulospinally mediated (Lockhart and Ting, 2007) and faster than cortically mediated agonist muscle activity (McKay et al., 2021). Consistent with the “shortening response” characteristic of PD, the destabilizing antagonist activity bursts driven by acceleration feedback were larger in PD than OA in forward perturbations (McKay et al., 2021). However, we found no difference in antagonist destabilizing components between OD and PD cohorts. Furthermore, we only found association of this component to balance ability in OAs based on clinical tests (Fig. 4*A*, red trace; Table 3). This difference in association between groups mirrors prior analysis of this same cohort that showed perturbation-evoked cortical activity did not differ between groups but was differently correlated between aspects of cognitive and balance ability (Payne et al., 2022). Similar to the prior finding, we found correlations of muscle activity to balance function among the OA group, but a lack of associations in PD, which may reflect more complex changes in the neural control of balance that occurs as PD progresses (Payne et al., 2022). Further, older adults with worse clinical balance had larger stabilizing responses to perturbation deceleration (Fig. 4*A*, green trace; Table 3), which could indicate that they are less able to take advantage of the platform stopping to return themselves to a stable standing state.

When comparing our reconstructions of agonist muscle activity from OAs with and without PD to previously published reconstructions from YAs (Boebinger et al., 2024), we found that OAs regardless of PD exhibit an LLR2 at smaller perturbation magnitudes than YAs. Unfortunately, the YA paradigm only had perturbations in a single direction while the OA and PD paradigm had perturbations in both directions, preventing direct comparisons. The YA group may have adapted their motor response to the perturbation paradigm (Welch and Ting, 2014), which can lead also to less cortical input (Jacobsen and Ferris, 2023, 2024; Refy et al., 2023; Liu et al., 2024). Nevertheless, our observation that OAs have more cortically mediated muscle activity at smaller disturbances is consistent with the increased activation of cortical areas observed in aging (Reuter-Lorenz and Cappell, 2008; Kang et al., 2022) and is in line with the hypothesis that cortical contributions to balance control increase with aging and impairment (Shumway-Cook et al., 1997; Brown et al., 1999; Woollacott and Shumway-Cook, 2002; Jacobs and Horak, 2007; Bayot et al., 2018; Stuart et al., 2018, 2019; Kahya et al., 2019).

Our hierarchical feedback model may thus index individual differences in the hierarchical control of balance without the need to measure brain activity. Perturbation-evoked muscle activity offers a more robust way to characterize cortical contributions to muscle activity at the millisecond-level temporal resolution of EMG using a few trials. In contrast, cortical engagement in a motor task is typically inferred based on changes in the motor behavior over a task-block when a simultaneous cognitive task (e.g., mental math) is introduced (Kahneman, 1973; Shumway-Cook et al., 1997; Wickens, 2002; Woollacott and Shumway-Cook, 2002; Watanabe and Funahashi, 2014; Ozdemir et al., 2016). Cortical engagement has also been assessed through optical measurements of cortical oxygen metabolism using functional near-infrared spectroscopy (fNIRS; Karim et al., 2012; Stuart et al., 2018, 2019; Pelicioni et al., 2019). However, fNIRS is subject to delays due to neurovascular coupling and can often only used to assess prefrontal oxygen metabolism as hair on the rest of the scalp prevents this technique from being used on areas over other neural structures (Khan et al., 2012; Scholkmann et al., 2014; Kassab et al., 2015; Yücel et al., 2017; Kim et al., 2024). In contrast, muscle activity reflects a more direct link to motor performance, as it reflects both a neurophysiological signal (i.e., motor neuron activity) and the biomechanical output necessary to generate movement.

In summary, we show that OAs, regardless of PD, have similar sensorimotor transformations underlying perturbation-evoked agonist activity as YAs. Our neuromechanical model can successfully dissociate perturbation-evoked agonist and antagonist muscle activity into hierarchical components using delayed CoM kinematic in OAs with and without PD. Comparisons of these components with those previously published in a YA cohort to literature suggest that the relative component magnitudes may differ with aging and impairment. Identifying hierarchical contributions to perturbation-evoked muscle activity may provide a means to assess individual difference in balance control for precision rehabilitation. Reactive balance paradigms could be used to quantify components of both agonist and antagonist muscle activity, thereby providing a metric of hierarchal contributions to balance control that could be tracked throughout disease progression and rehabilitation interventions on an individualized basis (Guadagnoli and Lee, 2004; Sawers et al., 2015). Additionally, the effect of assistive devices on an individual's control of balance could be inferred by the degree to which the balance control is cortically mediated while using the assistive device (Kim, 2025). Furthermore, our neuromechanical modeling technique has been extended to reconstruct joint torques, which would circumvent the need to record muscle activity (Jakubowski et al., 2025).

## Synthesis

Reviewing Editor: David Franklin, Technische Universitat Munchen Decisions are customarily a result of the Reviewing Editor and the peer reviewers coming together and discussing their recommendations until a consensus is reached. When revisions are invited, a fact-based synthesis statement explaining their decision and outlining what is needed to prepare a revision will be listed below. The following reviewer(s) agreed to reveal their identity: NONE.

Your manuscript has now been reviewed by two experts. While we all appreciate the scientific work and feel that the results will be of interest to a broad field, we have some concerns about the current version of the manuscript. In particular, we find some of the details in terms of the experimental work are missing, especially details on included and excluded trials. We also have some concerns regarding the statistics, and would appreciate some further details to motivate this study. I have combined the comments and concerns from both reviewers into a single decision letter below to explain our concerns and decision. We hope that you will use these comments to revise your manuscript, and look forward to your resubmission.

Specific Comments from the reviewers:

This nice paper translates previously validated models of neuromuscular feedback of balance recovery to older adults and a clinical group (individuals with PD). The findings reveal the existence of parallel feedback loops (with different input from hierarchical neural circuits), which can provide important insight into how age and neurological disease disturbs balance control. We appreciate the presentation of correlations between the clinical measures and the model results. The analysis is sound and we believe the results will be of interest to the broader field of neural control of movement.

Major comments:

It is stated that trials in which participants took a step were excluded-and that whole blocks of perturbations were excluded if there were less than 3 non-stepping trials. But I could not see any information on how many trials and conditions were excluded, and if this differed across participant groups? Also, is n=3 trials per perturbation size sufficient for determining reliable data? (I.e., it was not clear why this threshold was set.) It would also be useful to explicitly report the number of trials per condition (I believe it is n=8 per forward/backwards and perturbation size).

Describe the design and how many different combinations you measure, i.e. how many different trial-types were there in total (e.g., two perturbations directions (forward, backward), three perturbation magnitudes (small, medium and large) and so on...)

Given that a lot is made on the (borderline significant) correlations, it would greatly aid interpretation to plot these (especially considering the antagonist-stabilizing with minibest correlation is trending for the PD group as well, with p = 0.08). Are any techniques in place to statistically control for the large number of correlations conducted to avoid spurious findings?

"However, there was no significant difference in peak CoM excursion following a balance perturbation between OAs and individuals with PD for either forward (F(1, 33.8) = 2.88, p = 0.10) or backward perturbations (F(1, 34) = 0.36, p = 0.55)." Did you look at the CoM when left vs. right foot was forward? Did you look at differences for different sizes of perturbations?

I felt that the rationale for the study in the Introduction could be clearer; as the primary analysis does not allow you to test the effects of aging per se (as you do not have a younger healthy comparison group, only individuals with PD and matched older adults). Prior work from the group has also shown a lack of difference between older adults and individuals with PD with respect to the cortical response to perturbations (https://pubmed.ncbi.nlm.nih.gov/36043162/), so it is not clear why it is expected that there will be differences for cortical input to EMG responses? And how does the present work differ from prior work using similar models in PD? E.g. "The antagonist neuromechanical model also uses two feedback loops with different delays to reconstruct antagonist muscle activity and was developed in a prior study on a separate PD group (McKay et al. 2021)".

Minor comments:

Please include line numbers in the submission. This would have helped especially with the first submission.

Abstract: I would like a description of what was done. Which muscles were recorded? Were there actual participants or a model thereof?

In the introduction, when short and long latency responses are first introduced, it would be useful to provide the reader with some kind of ballpark idea of the latencies of these responses.

Is this a secondary analysis of data from Payne et al. 2021, 2022, 2023? If so, please make this explicit.

"If there were under 3 successful non-stepping trials averaged for a certain participant-condition pair, it was removed from analyses." Was there any difference in success rate between early and late blocks?

"Balance perturbations"-section: Please describe the ground position that the participants had. A description of the backward/forward positions would make it easier to understand what you did. Did you use the same type of starting position (i.e., right foot always forward? Or was it altered in a controlled manner? Or were the feet in anatomical ground position and Figures 1 and 2 are confusing me?)

Figure 1 would have benefitted from clearer labels. I had to read the legends a few times to understand that the middle panel is the hypothesised response in healthy older adults, whereas the right panel was for those with PD.

How long did the perturbation tests last? If exact times are not known, an approximation would be useful to include.

It might have been my bad eyesight, but I found the colour-schemes in figure 2 very hard to decipher.

Table 1; I presume the duration of PD is in years? Relatedly, it might be worth highlighting to readers unaware that MDS- UPDRSIII scores are higher = greater motor impairment (whereas minibest, higher = better balance function). I know it is in the main manuscript, but this can be added easily to the legend.

"(Forward: R2: t(34.5) = 11.2, p< 0.0001; VAF: t(34.7) = 11.0, p < 0.0001; Backward: (R2: t(35) = 6.6, p < 0.0001; VAF: t(35) =6.3, p < 0.0001)" This is just an example of where I see this. Why is there a difference in degrees of freedom between the forward and the backward models? Did you have to exclude trials, blocks or participants for some of the models? Did more forward trials end in participants taking a step forward or backward? Did this differ between the degrees of perturbation?

Table 2: "VAF = 0.72{plus minus}0.13". Why is the confidence interval of the VAF single loop so much higher compared to all of the other confidence intervals? (all the others are {plus minus}0.01-0.02)

"Forward: F(2, 6.38) = 1.59, p = 0.21; Backward: F(2, 68) = 0.39, p = 0.68)" Do you really mean 6.38 in the first test here? If it is correct, why is the denominator degrees of freedom so different between the models? Else this suggests that you used different types of test for the different models, which would then need to be justified. However, the previous lines suggest that it is a typo and should be 63.8. In relation to this, were there any differences between the bilateral recordings? Do you obtain the same type of results by splitting the data into left leg vs. right leg?

It is stated (in Discussion): "Furthermore, we show that the LLR2 component increases with balance difficulty and is larger in OAs than YAs". But this was not formally tested??

As a stylistic choice, it seemed that the Discussion suddenly ended without a clear conclusion.
